# The impact of family functioning on college students' differentiation of self: a serial mediation analysis and gender differences

**DOI:** 10.3389/fpsyg.2026.1911303

**Published:** 2026-09-11

**Authors:** Wei Luo, Jie Gao, Haixia Zhao, Meng Zhang, Wenjuan Wang

**Affiliations:** 1 Student Affairs Department, Xi'an University of Science and Technology, Xi'an, Shaanxi, China; 2 Department of Primary Education, Shaanxi Provincial Youth School, Xi'an, Shaanxi, China; 3 School of Economics and Management, Huzhou Normal University, Huzhou, Zhejiang, China

**Keywords:** differentiation of self, family functioning, gender difference, loneliness, psychological security, serial mediating effect

## Abstract

**Objective:**

This study investigated the relationship between family functioning and college students' differentiation of self, with a particular focus on the serial mediation of loneliness and psychological security, and further examined cross-gender invariance and gender moderation effects in this mediation model.

**Method:**

A questionnaire survey was administered to 5,485 first-year undergraduate students recruited from three ordinary universities, using the Family Functioning Scale, the Emotional and Social Loneliness Scale, the Safety Scale, and the Differentiation of Self Scale. Data were analyzed using SPSS 24.0, the PROCESS macro, and Mplus 8.3. Analytical procedures included correlation analysis, serial mediation effect testing, multi-group structural equation modeling (SEM), and gender moderation analysis.

**Results:**

(1) College students' differentiation of self was generally at a moderate-to-high level, with significant gender differences such that male students scored higher than their female counterparts. (2) Family functioning significantly and negatively predicted differentiation of self, and this effect was partially mediated by loneliness and psychological security, both independently and sequentially through a serial mediation pathway. (3) Multi-group SEM confirmed cross-gender measurement and structural invariance of the proposed model, indicating overall stability of the mechanism; however, significant gender moderation effects were observed on specific indirect pathways.

**Conclusion:**

Family functioning predicts differentiation of self through both direct and indirect effects. These findings elucidate the internal psychological mechanisms and gender-specific characteristics underlying this relationship, offering theoretical and empirical grounds for gender-tailored mental health education and family-based intervention strategies for college students.

## 1 Introduction

The college stage represents a critical developmental period during which individuals form an independent personality, progressively attain physical and psychological autonomy, and gradually free themselves from emotional dependence on their parents. Throughout this process, students are confronted with multiple challenges, including academic competition, the reconstruction of interpersonal relationships, and the exploration of self-identity. As a pivotal period and context for differentiating from the family of origin (Bowen, 1978), college students' Differentiation of Self (DOS) entails a dual complexity: they must not only break free from the inertial constraints of the family emotional system (Kerr and Bowen, 1988) but also establish autonomy in navigating novel challenges such as academic competition and peer pressure.

### 1.1 The multidimensional value and developmental mechanism of differentiation of self

Differentiation of Self is a core construct introduced by Bowen (1976) to describe individuals' capacity to distinguish between emotion and intellect, and between self and others. Specifically, it refers to the ability to clearly define one's self-identity without being overwhelmed by emotional reactivity, while simultaneously maintaining close yet stable interpersonal relationships (Peleg and Boniel-Nissim, 2024). The construct comprises four dimensions: emotional reactivity, “I” position, emotional cutoff, and fusion with others (Wu, 2013; Choi and Murdock, 2017). Differentiation of Self operates at two interrelated levels. At the intrapsychic level, highly differentiated individuals are capable of maintaining rational judgment even in the presence of intense emotional arousal (Peleg and Boniel-Nissim, 2024) and adhering to their own convictions under external pressure (Wu et al., 2022); by contrast, those with low differentiation exhibit emotional dominance over rationality, manifesting impulsive reactions to situational triggers (Krycak et al., 2012; An and Man, 2018). At the interpersonal level, highly differentiated individuals can sustain emotional connectedness with others (Calatrava et al., 2020) while simultaneously preserving independent values and personal boundaries (An and Chen, 2015); conversely, less differentiated individuals tend to experience emotional fusion and blurred boundaries with others, or alternatively, resort to emotional cutoff by avoiding intimate relationships.

Differentiation of self develops within the context of family emotional relationships and manifests across both intrapsychic and interpersonal domains, with notable individual and cultural variations (Narayanan and Sriram, 2025; Peleg and Messerschmidt-Grandi, 2019; Hocaoglu and Işik, 2023). Within the Chinese cultural context, college students rarely express anger openly in public; even when experiencing intense internal emotional conflicts, they tend to restrain negative emotions in the presence of family members and peers (An et al., 2012a,b). Higher levels of differentiation of self among college students are associated with lower levels of depression and anxiety (Wu et al., 2022; Peleg and Boniel-Nissim, 2024), as well as reduced academic stress (Wu, 2013), campus maladjustment (Süloglu and Güler, 2021), family conflict (Jankowski et al., 2013), and romantic relationship distress (Cao and An, 2016). Conversely, psychological distress among individuals with low differentiation of self may stem from vulnerable narcissistic tendencies and maladaptive perfectionism (Hosack et al., 2023). Owing to deficits in emotion regulation and blurred self-boundaries (Krycak et al., 2012), such individuals are more likely to enter a negative anxiety-avoidance cycle and experience a range of psychological difficulties (Wu et al., 2022), including depressive symptoms and somatic complaints (Choi and Murdock, 2017). They also tend to adopt maladaptive coping strategies, such as procrastination (Guo et al., 2021), smartphone dependency (Ercengiz et al., 2024), internet addiction (Li et al., 2023), and substance abuse (Krycak et al., 2012), and to form inauthentic interpersonal relationships through people-pleasing and emotional dependency (Bartle-Haring et al., 2019). Clinical practice further suggests that enhancing individuals' differentiation of self contributes to improvements in intimate relationships and overall physical and mental health (Peleg and Boniel-Nissim, 2024).

According to Bowen's family systems theory, gender socialization shapes distinct patterns of self-boundary formation for males and females. From early childhood, males are socialized to pursue independence and restrain emotional expression, which predisposes them to develop clearer interpersonal boundaries and lower levels of emotional fusion with others. In contrast, females are socialized to prioritize emotional connectedness and relationship maintenance, rendering them more susceptible to being influenced by others' emotions. This theoretical inference is corroborated by a substantial body of domestic empirical research. For instance, Yao et al. (2011), drawing on a sample of over one thousand college students, found that male undergraduates scored significantly higher than their female counterparts on both the total scale and all subscales of the Differentiation of Self Inventory. Similarly, Wu (2010) reported that male college students scored significantly higher than females on the emotional reactivity, “I” position, and fusion with others subscales, as well as on the total scale of differentiation of self. These converging findings consistently support the existence of gender differences in self-differentiation among Chinese college students.

### 1.2 The influence of family functioning on college students' differentiation of self

Family functioning refers to the comprehensive operational capacity of the family system to fulfill its core tasks and provide a healthy developmental environment—physically, psychologically, and socially (Grandi et al., 2007). It is a crucial protective factor for maintaining healthy family structures, facilitating emotional growth, and enhancing quality of life (Krün et al., 2025). The essence of family functioning lies not in its external structure, but rather in the patterns of family organization, communication processes, and the multigenerational life-cycle patterns and belief systems (Walsh, 1994). The fundamental mission of the family is to serve as a “developmental laboratory,” addressing multiple needs including material provision, emotional nurturing, personality development, and crisis coping (Skinner et al., 2000). Family functioning encompasses seven basic dimensions: task accomplishment, role performance, communication, affective expression, involvement, control, and values and norms. Within the family system, differentiation of self is conceptualized as the capacity to balance emotional fusion and autonomy (Skowron and Friedlander, 1998)—that is, to maintain emotional connectedness while simultaneously establishing clear self-boundaries (Wu and Wang, 2011). Differentiation of self is regarded as one of the most critical personality traits for individual psychological maturity, as well as an essential developmental goal for family members (Kerr, 1984). The family constitutes the most direct and enduring developmental context for shaping individual differentiation of self. As the primary agent of socialization, the family provides the most fundamental interpersonal interaction settings (Kerr and Bowen, 1988). Proximal family relationship processes serve as the most powerful predictors of individual self-development (Zhao et al., 2026), particularly during late adolescence and emerging adulthood—a period when individuals gradually reduce their dependence on parents yet continue to maintain close emotional ties with their families.

An individual's level of differentiation of self-influences their psychosocial functioning (Ercengiz et al., 2024) and is jointly shaped by multigenerational emotional patterns, family emotional projection processes, and family triangulation within the family of origin (Wu, 2013). Families characterized by low differentiation tend to exhibit coping patterns such as emotional fusion, emotional over-reactivity, and emotional cutoff (Bowen, 1978; Peleg and Boniel-Nissim, 2024). In family environments that are respectful, open, moderately intimate, and supportive, individuals are able to express their own views (Peleg et al., 2018) and attain an appropriate degree of autonomy (Skowron et al., 2010), regulating their emotions through rational cognitive processes rather than becoming immersed in states of emotional fusion. Healthy family functioning has been shown to promote individuals' differentiation of self (Rodríguez-González et al., 2019), facilitating the establishment of clear psychological boundaries (Watermeyer and Swartz, 2008), emotional stability (Shapero et al., 2016), and independent judgment, while reducing rejection sensitivity (Skowron et al., 2010), thereby elevating differentiation of self. Conversely, frequent family conflict (Van Eldik et al., 2020), triangulation in family relationships (McCauley et al., 2021), excessive parental emotional over-involvement, and inconsistent parenting styles (Dwairy, 2008) can all impede the development of differentiation of self. Chaotic or overly fused family interaction patterns tend to blur the emotional boundaries between individuals and their families, giving rise to emotional conflict, cognitive confusion, and excessive emotional fusion (Chung and Gale, 2009), as well as externalizing behavior problems (Forbes et al., 2017), alcohol use (Laghi et al., 2012), and depressive symptoms (Tamplin and Gooyer, 2001).

### 1.3 The mediating effect of loneliness

Loneliness, as a negative emotional experience, arises when individuals perceive deficiencies in either the quantity or quality of their social networks (Jiang et al., 2017). First-year undergraduate students commonly encounter multifaceted challenges when adapting to new learning environments, with loneliness emerging as a salient psychological feature during this transitional period (Jiang et al., 2017). The close teacher-student and peer relationships that students previously maintained based on geographic proximity are disrupted upon entering university, whereas new support systems rooted in shared interests and values have yet to be fully established (Jin, 2019). Online-mediated communication is an indispensable part of college students' social life. Even interactions with nearby peers frequently take place via social-media platforms. Such patterns may reduce deep-level interpersonal engagement and result in superficial social contact without genuine emotional bonding, which further contributes to subjective loneliness (Livingstone, 2018). This social context helps explain the high prevalence of loneliness among contemporary university students. Modern communication technologies may partially sustain existing interpersonal ties; however, long-distance and predominantly online interactions often lack the depth, emotional richness, and immediate feedback characteristic of face-to-face communication (Livingstone, 2018). Over-reliance on online social networking may even lead to the deterioration of real-world social skills and increased social isolation (Jiang et al., 2017).

The broader social context provides a fertile ground for loneliness among college students. According to (Li 2012), approximately 45% of college students experience moderate to high levels of loneliness (Li, 2012). Contemporary societal values emphasize individualization and self-actualization, with young people increasingly inclined to pursue personal expression and individual goals (Liu and Yu, 2025). This cultural orientation weakens the sense of belonging, making individuals more vulnerable to feelings of isolation when facing challenges (Datu, 2017). Within university settings, the lack of in-depth communication among dormitory mates and classmates often reduces them to relationally distant “strangers” (Zhang, 2015). For most college students, loneliness tends to persist for 2–3 months; however, those with maladaptive adjustment patterns may experience more enduring and severe loneliness (Yan and Yao, 2025). Furthermore, loneliness has been shown to weaken individuals' perceived support from both friends and family (Kulari, 2025), and is significantly associated with a range of psychological and behavioral problems among college students, including depression, anxiety, social avoidance, sleep disturbances, and even substance abuse (Hawkley and Cacioppo, 2010; Lim et al., 2020).

Loneliness may play a significant mediating role in the relationship between family functioning and individual differentiation of self. Dysfunctional family environments characterized by low emotional expression and high conflict may impede the development of self-differentiation, rendering individuals less capable of coping independently with challenges after leaving their family of origin, which in turn exacerbates feelings of loneliness (An et al., 2012a,b). Conversely, adequate family support serves as a crucial buffer against loneliness (Hutten et al., 2021). The emotional experiences derived from family interactions are internalized as subjective judgments regarding one's social connectedness. Once activated, the experience of loneliness may further interfere with individuals' emotional regulation, boundary establishment, and interpersonal processing patterns (Kulari, 2025). Persistent loneliness renders individuals more susceptible to emotional fusion or emotional cutoff, impairing their capacity to rationally distinguish their own emotions from others' expectations, and ultimately inhibiting the enhancement of self-differentiation levels.

### 1.4 The mediating effect of psychological security

Psychological security may play a significant mediating role between family functioning and differentiation of self among university first-year undergraduate students. Psychological security refers to an intrinsic need for a stable and predictable environment; when individuals perceive their surroundings as controllable and feel capable of anticipating and managing potential threats, they experience a psychological state characterized by a sense of strength and certainty (Ren, 2024). From a humanistic perspective, Maslow's hierarchy of needs positions security as the second most fundamental need, subordinate only to physiological requirements, encompassing a dual desire for environmental stability and emotional acceptance.

The construction of psychological security primarily rests upon two key dimensions: (1) environmental predictability (Yang et al., 2025), and (2) self-efficacy in coping (Ren, 2024). For university first-year undergraduate students, environmental changes—such as studying away from home and cultural differences—can readily undermine their sense of security, while the as-yet-unestablished stable interpersonal network further amplifies this uncertainty. In this context, the family, serving as the primary external support system (Wen et al., 2021), plays a critical role in determining whether first-year undergraduate students can obtain sufficient security buffer, through the quality of family functioning, including emotional responsiveness and conflict resolution abilities. Drawing upon Bowen's Family Systems Theory, family functioning—encompassing communication patterns, emotional support, and boundary clarity—shapes members' psychological security, defined as the sense that “I can express myself authentically without fear of being devalued or abandoned,” which in turn influences their level of differentiation of self.

It is noteworthy that psychological security and loneliness are dynamically interrelated. Empirical evidence indicates that psychological security significantly and negatively predicts social avoidance and loneliness among college students (Yu et al., 2022), with family functioning serving as the starting point of this protective pathway (Yang et al., 2025). On this basis, the present study conceptualizes loneliness and psychological security as mediating variables in the relationship between family functioning and differentiation of self. The underlying mechanism posits that stable family support enhances individuals' sense of environmental control and self-efficacy, thereby facilitating exploration and connectedness during the differentiation process and contributing to the formation of a healthy autonomous personality. This study aims to explore the factors influencing differentiation of self among college students and proposes the following hypotheses:

Hypothesis 1: College students' self-differentiation will be at a moderate-to-high level, with significant gender differences such that male students will report significantly higher overall levels of self-differentiation than their female counterparts.
Hypothesis 2: Family functioning will positively predict college students' self-differentiation, such that individuals with higher levels of family functioning will exhibit higher levels of self-differentiation.
Hypothesis 3: Loneliness and psychological security will serve as serial mediators in the relationship between family functioning and college students' self-differentiation. Specifically, loneliness will exert a negative mediating effect, whereas psychological security will exert a positive mediating effect.

## 2 Methods

### 2.1 Participants

Data collection was conducted from September to November 2025. Using convenience sampling, we recruited first-year undergraduate students from three ordinary universities across the central-western region of China. With the approval and cooperation of the student affairs departments, psychological teachers served as examiners and administered the survey in classroom settings and during evening self-study sessions, with each session lasting approximately 20 min. Students completed the questionnaires anonymously and truthfully after providing informed consent online. A total of 5,769 students completed the survey. The inclusion criteria were: (1) full-time undergraduate students; and (2) students who had signed the informed consent form and agreed to participate in the survey. The exclusion criteria were: (1) respondents who demonstrated obviously careless or patterned responding; and (2) failure to correctly answer the instructed response item embedded in the questionnaire (i.e., “Please select ‘comparatively inconsistent' for this item”). A total of 284 invalid responses were excluded. The final sample consisted of 5,485 participants, with a mean age of 18.04 years (SD = 0.67), with ages ranging from 17 to 20 years. Among them, 3,483 (63.5%) were male and 2,002 (36.5%) were female; 1,766 (32.2%) were only children and 3,719 (67.8%) were not.

### 2.2 Measures

#### 2.2.1 Family Assessment Device (FAD)

*The Family Assessment Device* (FAD) was developed on the basis of *the McMaster Model of Family Functioning* (MMFF), which determines the measurement scope of the scale (Epstein et al., 1983). Focusing on interactions and systemic properties within the family system, the scale is used to collect information across various domains of overall family functioning (Su and Duan, 2008). It consists of 60 items rated on a 4-point Likert scale, with several items reverse-scored. Higher total scores indicate lower family functioning. The scale has demonstrated good reliability and validity. It comprises seven subscales: Problem Solving (PS), Communication (CM), Roles (RL), Affective Responsiveness (AR), Affective Involvement (AI), Behavior Control (BC), and General Functioning (GF). In the present study, the Cronbach's α coefficient for the full scale was 0.91.

#### 2.2.2 Emotional vs. Social Loneliness Scales (ESL)

*The Emotional vs. Social Loneliness Scales* were developed by Wittenberg and Reis (1986) based on the theoretical framework proposed by Weiss (1973). The scale consists of 10 items rated on a 5-point Likert scale, with higher scores indicating more severe loneliness. It comprises two subscales: (a) Emotional Loneliness (5 items), which pertains to the evaluation of various friendships and arises from a lack of satisfying friendships; (b) Social Loneliness (5 items), which pertains to the evaluation of various intimate relationships and results from a lack of satisfying romantic relationships. In the present study, the Cronbach's α coefficient for the full scale was 0.87.

#### 2.2.3 Security Questionnaire (SE)

*The Security Questionnaire* (SQ) was developed by An and Cong (2003). This self-rating scale consists of 16 items rated on a 5-point Likert scale, comprising two factors. Higher scores indicate a higher level of psychological security. The scale includes two subscales: (a) Interpersonal Security Factor (8 items), which primarily reflects individuals' security experiences during interpersonal interactions; (b) Certainty and Control Factor (8 items), which primarily reflects individuals' sense of predictability, certainty, and control over life. In the present study, the Cronbach's α coefficient for the full scale was 0.89.

#### 2.2.4 Differentiation of Self Scale (DOS)

*The College Student Differentiation of Self Scale*, revised by Wu and Wang (2011), was adopted in this study. The scale consists of 27 items rated on a 6-point Likert scale, with 22 items reverse-scored. Total scores are calculated, and higher scores indicate a higher level of differentiation of self. The scale comprises four dimensions: Emotional Reactivity, I-Position, Emotional Cutoff, and Fusion with Others. In the present study, the Cronbach's α coefficient for the full scale was 0.85, and the Cronbach's α coefficients for the four subscales were 0.84, 0.81, 0.82, and 0.91, respectively.

In addition, various sociodemographic data were collected, including gender, age, and only-child status, ethnicity, majors.

### 2.3 Procedures

Group-based data collection was organized on a class basis. With approval obtained from the Mental Health Education Ethics Committee and the student-affairs departments of the participating universities, first-year undergraduate students from three ordinary Chinese undergraduate universities were recruited as participants. All participants provided electronic informed consent online prior to completing the survey. The questionnaire was administered entirely online, and participants were required to respond to all items without leaving any blanks. Validity check items were embedded in the questionnaire. Responses that failed to follow standard answering rules were eliminated, and a total of 284 invalid questionnaires were excluded. Statistical analyses were conducted using SPSS 24.0 (Muthén), the PROCESS macro developed by Hayes, and Mplus 8.3 (Muthén). Descriptive statistics and Pearson correlation analyses were first compute using SPSS 24.0. The hypothesized serial mediation model was then tested with Mplus 8.3. In addition, the PROCESS macro was employed within SPSS to incorporate gender as a control variable in the serial mediation framework.

All participants took part in the survey voluntarily with informed consent. SPSS 24.0 and Mplus 8.3 were used for statistical data analysis. Firstly, SPSS 24.0 was adopted to analyze the distribution of all variables and calculate correlation coefficients. Secondly, Mplus 8.3 was used to test the hypothesized serial mediating effects while controlling for relevant variables.

## 3 Results

### 3.1 Common method bias test

Data collected via self-report measures may be susceptible to common method bias (CMB). Therefore, multiple procedural controls were implemented prior to data collection to mitigate this potential bias. Specifically, all test administrators received standardized training and followed unified administration procedures. Participants were explicitly informed that all data collected would be used solely for scientific research purposes and that their responses would remain anonymous. Several reverse-scored items were embedded in the questionnaire, and validity check items were included to identify careless responses. Questionnaires that failed to meet the prescribed response criteria were excluded from further analysis.

After data collection, Harman's single-factor test was conducted as a statistical control. An unrotated principal component factor analysis was performed on all items of the measured variables. The results showed that the first factor accounted for 25.81% of the total variance, which was below the commonly accepted threshold of 40%. This finding suggests that common method bias did not pose a serious threat to the validity of the present dataset.

### 3.2 Descriptive statistics and correlation analysis of variables

As shown in Table 1, college students reported moderate-to-high mean scores on psychological security (M = 4.11, SD = 0.89) and Differentiation of Self (M = 3.87, SD = 0.80), whereas their mean scores on family functioning (M = 1.88, SD = 0.31) and emotional vs. social loneliness (M = 2.48, SD = 0.64) were relatively low. Pearson correlation analyses revealed that family functioning was significantly negatively correlated with both psychological security (*r* = −0.59, *p* < 0.001) and Differentiation of Self (*r* = −0.58, *p* < 0.001), and significantly positively correlated with loneliness (*r* = 0.44, *p* < 0.001). Psychological security was significantly negatively correlated with loneliness (*r* = −0.51, *p* < 0.001). Differentiation of Self exhibited significant negative correlations with family functioning (*r* = −0.58, *p* < 0.001) and loneliness (*r* = −0.48, *p* < 0.001), and a significant positive correlation with psychological security (*r* = 0.84, *p* < 0.001). These correlational patterns provided preliminary support for subsequent hypothesis testing.

**Table 1:** Descriptive statistics and correlation matrix of all variables (*N* = 5,485).

Variables	*M*	*SD*	1	2	3	4	5
1.Gender	1.41	0.48					
2.only-child status	1.32	0.44	−0.30				
3.Family functioning	1.88	0.31	0.04^*^	−0.07^*^			
4.Emotional vs. Social loneliness	2.48	0.64	−0.01	−0.03^*^	0.44^***^		
5.Security	4.11	0.89	0.07^*^	0.03^*^	−0.59^***^	−0.51^***^	
6.Differentiation of self	3.87	0.80	0.12^**^	−0.02^*^	−0.58^***^	−0.48^***^	0.84^***^

^*^p < 0.05, ^**^p < 0.01, ^***^p < 0.001. For the family-functioning scale, higher scores indicate lower family functioning.

Independent-samples *t*-tests further indicated that male students scored significantly higher than female counterparts on psychological security (M_male_ = 4.05, M_female_ = 3.92, *p* < 0.01) and Differentiation of Self (M_male_ = 3.95, M_female_ = 3.75, *p* < 0.01). With respect to the subscales of Differentiation of Self, significant gender differences were observed for emotional reactivity, “I” position, and fusion with others, with male students scoring significantly higher than female students on all three dimensions (*p*s < 0.001). However, the gender difference on the emotional cutoff subscale did not reach statistical significance (*p* > 0.05; see Table 2).

**Table 2:** Gender differences in subscales of differentiation of self: independent-samples *t*-tests (M ± SD).

Variables	Total	Male	Female	*t*
Emotional reactivity	3.54 ± 1.08	3.67 ± 1.11	3.34 ± 0.95	9.69^***^
“I” position	4.31 ± 0.98	4.41 ± 1.03	4.16 ± 0.84	8.01^***^
Emotional cutoff	4.25 ± 1.01	4.23 ± 1.05	4.27 ± 0.87	−1.12
Fusion with others	3.61 ± 1.01	3.69 ± 1.05	3.47 ± 0.89	6.74^***^

^***^p < 0.001.

### 3.3 The relationship between family functioning and differentiation of self: a test of serial mediating effect

To effectively control for measurement error, structural equation modeling (SEM) was adopted to test the multiple mediation effects. After controlling for gender, age, ethnicity, and academic major, the total effect of family functioning on Differentiation of Self was examined. The results revealed a significant path coefficient (γ= 0.14, *t* = 16.47, SE = 0.01, *p* < 0.001).

**Figure 1:**
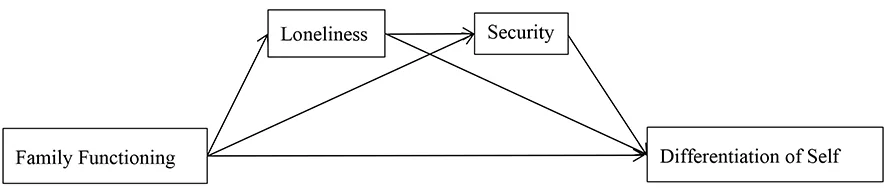
Hypothesized mediation model.

**Figure 2:**
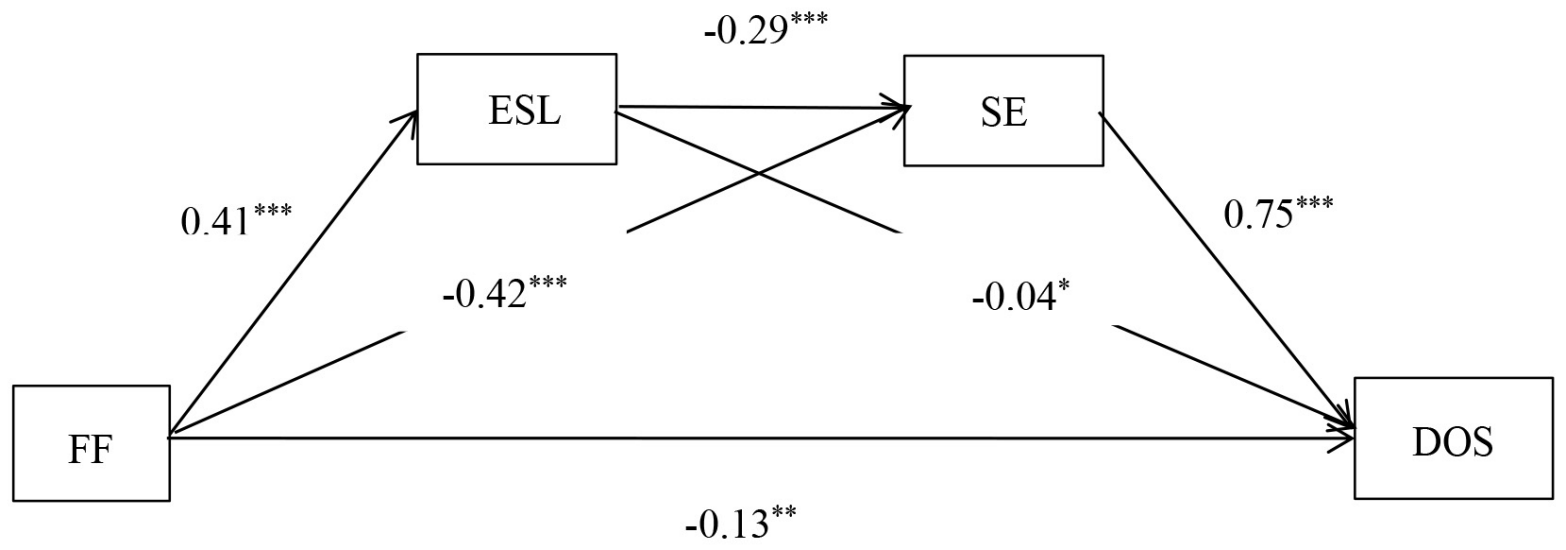
Serial mediating model (*N* = 5,485). *p < 0.05, **p < 0.01, ***p < 0.001; the same hereinafter. All data represent standardized path coefficients. FF, Family Functioning; DOS, Differentiation of Self; ESL, Emotional vs. Social Loneliness; SE, Security. For the family-functioning scale, higher scores indicate lower family functioning.

Loneliness and psychological security were subsequently incorporated into the model as mediating variables. Comparisons among the two separate single-mediator models and the dual-mediator model indicated that the serial mediation SEM achieved superior data fit. As presented in Figures 1, 2, all path coefficients were statistically significant (*p* < 0.01), and the model exhibited acceptable fit indices: χ^2^/*df* = 3.59, CFI = 0.98, TLI = 0.96, RMSEA = 0.08, SRMR = 0.03. The model demonstrated that family functioning exerted both a direct effect on Differentiation of Self and indirect effects through loneliness and psychological security as serial mediators.

The mediation effects encompass three pathways: loneliness mediates the relationship between family functioning and psychological security; psychological security mediates the relationship between family functioning and differentiation of self; and loneliness and psychological security serve as serial mediators between family functioning and differentiation of self. Bias-corrected non-parametric percentile bootstrapping with 5,000 resamples was employed to test the mediation effects and estimate 95% confidence intervals. An indirect effect was considered significant if the 95% confidence interval did not include zero. The results indicated that loneliness mediated the relationship between family functioning and psychological security, psychological security mediated the relationship between family functioning and differentiation of self, and the serial mediation pathway through loneliness and psychological security was also significant (see Table 3). While this indirect path was statistically significant (95% CIs did not include zero), its standardized point estimate was negligible (−0.023), suggesting little substantive contribution. Such significant yet weak effects are likely driven by the large sample size of the current study (*N* = 5,498).

**Table 3:** Mediating effect sizes and estimates.

Path	Standardized effect estimate	95% CI	

		Lower	Upper
FF → DOS	−0.117	−0.134	−0.100
FF → ESL → DOS	−0.023	−0.033	−0.018
FF → SE → DOS	−0.347	−0.405	−0.366
FF → ESL → SE → DOS	−0.110	−0.133	−0.110
Total effect of FF on DOS	−0.597	−0.683	−0.536

The results are based on bias-corrected 95% confidence interval estimation. FF, Family Functioning; DOS, Differentiation of Self; ESL, Emotional vs. Social Loneliness; SE, Security. For the family-functioning scale, higher scores indicate lower family functioning.

### 3.4 Multi-group structural equation modeling

To examine cross-gender measurement invariance of the serial mediation model, a series of nested multigroup structural equation models were progressively constructed, including configural invariance, metric invariance, and scalar invariance. The fit indices for the three nested models indicated that CFI ranged from 0.922 to 0.929, TLI ranged from 0.915 to 0.924, both exceeding the recommended threshold of 0.90; RMSEA ranged from 0.088 to 0.095, and SRMR ranged from 0.093 to 0.099 (see Table 4). By imposing equality constraints on factor loadings across groups based on the baseline model, the metric invariance model was obtained, with no substantial deterioration in model fit. This suggests that the latent variables satisfy cross-gender metric invariance, implying that the scale items hold consistent measurement meanings across male and female college student groups, thereby justifying cross-group comparisons of path coefficients. After further constraining structural paths to be equal across groups, the model fit did not significantly decrease, providing preliminary support for scalar invariance.

**Table 4:** Fit indices of competing multi-group models.

Model	χ^2^	df	χ^2^/df	*Δχ* ^2^	Δdf	CFI	TLI	RMSEA	SRMR
Model 1: Configural baseline	5478.36	190	28.83	–	–	0.922	0.915	0.088	0.093
Model 2: Metric invariance	6640.32	193	34.41	1161.96	3	0.926	0.918	0.090	0.094
Model 3: Structural invariance	7340.97	196	37.45	700.65	3	0.929	0.924	0.095	0.099

Given that scalar invariance testing imposes constraints at the overall model level and has limited sensitivity to subtle differences in specific pathways, a moderated serial mediation model was employed to identify potential gender heterogeneity within the serial mediation pathways. To examine the moderating role of gender in the serial mediation model, we utilized PROCESS Macro to construct a moderated serial mediation model, with gender as the moderator, dimensions of family functioning as independent variables, and differentiation of self and its sub-dimensions as dependent variables. The results are as follows: (1) when emotional responsiveness and behavioral control served as independent variables, the serial mediation model exhibited distinct gender moderation patterns. When emotional responsiveness predicted overall differentiation of self, the overall model was significant (*F* = 2,533.25, *p* < 0.001), accounting for 76.41% of the variance. Gender significantly moderated the direct path from emotional responsiveness to differentiation of self, whereas the mediating pathways were not significantly moderated by gender. When behavioral control predicted overall differentiation of self, gender significantly moderated the serial mediation pathway of behavioral control → loneliness → psychological security → differentiation of self, with an index of moderated mediation of −0.7007 [95% CI [−1.0267, −0.3628]]. (2) When the dependent variable was specified as the self-position and emotional cutoff sub-dimensions of differentiation of self, respectively, the serial mediation effects exhibited differentiated gender heterogeneity. With self-position as the dependent variable, the overall model was significant (*F* = 313.00, *p* < 0.001), explaining 28.58% of the variance. The interaction terms of family functioning, loneliness, psychological security, and gender were all significant, indicating overall gender heterogeneity in the complete serial mediation pathway leading to self-position. With emotional cutoff as the dependent variable, the overall model was significant (*F* = 313.00, *p* < 0.001), explaining 57.14% of the variance. Only the interaction terms of loneliness, psychological security, and gender were significant, suggesting that the gender moderation effect was present only in the latter half of the serial mediation pathway leading to emotional cutoff.

## 4 Discussion

### 4.1 First-year undergraduate students demonstrate a relatively high level of differentiation of self

The research data indicate that contemporary university first-year undergraduate students as a whole demonstrate a relatively high level of differentiation of self. Male students scored significantly higher than their female counterparts on the subdimensions of emotional reactivity, I-position, and fusion with others, indicating that male college students possess higher overall differentiation of self (Hypothesis 1 supported). This finding aligns with prior empirical evidence (Wu, 2010). This relatively high level of differentiation of self suggests that, during the critical transitional phase from family environment to independent campus life, first-year undergraduate students have already developed foundational capacities for emotional regulation, independent thinking, and interpersonal boundary construction. These capacities enable them to navigate multiple challenges—including academic pressure, interpersonal adjustment, and self-identity reconstruction—with comparative ease, thereby laying a solid psychological foundation for their adaptation to university life. The observed gender differences may be attributed to distinct patterns of emotional processing and interpersonal boundary construction shaped by gender-specific socialization processes. Influenced by indigenous cultural norms, female students are more likely to assume the role of emotional anchors within the family and tend to take on the responsibility of regulating parental emotions, which may give rise to a form of “pseudo-maturity” (Li et al., 2023). In contrast, male students, socialized to be more emotionally restrained and judgmentally independent, are better able to maintain clear interpersonal boundaries.

The serial mediation model further confirmed heterogeneity in gender moderation effects. When self-position served as the outcome variable, the complete serial pathway through which family dysfunction affects self-position via loneliness and psychological security exhibited significant gender differences, indicating that the intensity and sensitivity of the impact of family-interaction-derived interpersonal boundary cognitions vary between male and female students. This suggests that gender-specific intervention strategies are needed to enhance individuals' levels of self-position. When emotional cutoff served as the outcome variable, gender only moderated the latter half of the serial pathway, namely loneliness → psychological security → emotional cutoff. Insufficient family support can trigger loneliness in both male and female students; however, the underlying mechanisms and risk levels involved in the transition from loneliness to emotional cutoff differ by gender. Psychological security may serve as a critical intervention target for ameliorating emotional cutoff among college students.

The moderated serial mediation model further confirmed that the predictive patterns of different dimensions of family functioning on differentiation of self among college students exhibit significant gender differentiation. Specifically, when emotional responsiveness of family functioning served as the predictor variable, the model explained 76.41% of the variance, rendering it the core factor predicting overall differentiation of self among college students. The indirect effects of the serial mediation pathway for this dimension did not differ significantly by gender, suggesting that the psychological transmission process—whereby family emotional stimuli trigger individual emotional fluctuations, which in turn induce loneliness and undermine psychological security—operates consistently across genders. However, the direct effect of emotional responsiveness on differentiation of self was significantly moderated by gender, indicating that equivalent levels of family emotional responsiveness exert differential direct effects on differentiation of self between male and female students. When behavioral control of family functioning served as the independent variable, the serial mediation effect was significantly moderated by gender. The negative index of moderated mediation indicated that, under the same level of family behavioral control, the magnitude of this serial mediation effect differed between male and female college students, reflecting notable gender differences in psychological responsiveness to family behavioral control patterns.

### 4.2 The predictive role of family functioning in differentiation of self among first-year undergraduate students

Correlation analysis reveals a moderate negative correlation between family functioning and differentiation of self. This indicator negatively predicts college students' level of differentiation of self, which reflects that better family functioning correlates with higher levels of differentiation of self among college students (Hypothesis 2 is supported).This indicator negatively predicts college students' level of differentiation of self, which reflects that better family functioning correlates with higher levels of differentiation of self among college students (Hypothesis 2 is supported).Correlation analyses suggest that family functioning is associated with individuals' differentiation of self via material and psychological support pathways. Families with high cohesion and flexible adaptive communication tend to provide a psychological “secure base” for college students, which is linked to greater confidence in expressing personal thoughts and emotions (Rodríguez-González et al., 2019). Consistent with this prior evidence, the current data shows that students from positive family functioning backgrounds tend to report higher differentiation of self.Family interaction patterns are correlated with the formation of individuals' interpersonal schemas, and are associated with the development of well-bounded, healthy close relationships in later life. In contrast, families marked by frequent conflicts and insufficient emotional communication tend to correspond with lower self-differentiation levels, alongside patterns of intergenerational emotional fusion. From a cultural perspective, social emphasis on academic achievement is correlated with slower development of self-differentiation among local college students. In the Chinese cultural context, children often bear the emotional burden of stabilizing family harmony. Under the influence of traditional filial piety norms, many young adults report a pattern of “pseudo-independence”: they demonstrate autonomous behaviors externally yet maintain emotional fusion with their families internally (Li et al., 2023).

Differentiation of self is a continuous developmental task that unfolds throughout the lifespan. Individuals may enhance their level of differentiation of self by deconstructing intergenerational emotional bonding patterns and reducing family triangulation, thereby alleviating anxiety arising from emotional fusion. According to Bowen's Family Systems Theory, families with low levels of differentiation tend to entangle children in marital conflicts, assigning them the role of an “emotional buffer,” which in turn inhibits the development of individual autonomy (Chung and Gale, 2009). Through intrinsic psychological organization and self-regulation, individuals achieve self-integration and coordinate the relationship between self and others. The external environment can either facilitate or hinder individual development, and the family, as a primary source of social support for college students, exerts a non-negligible influence on their psychological development.

Cultural context moderates the process through which family functioning affects differentiation of self. Within the Chinese sociocultural context, the general public places great emphasis on family identity, family harmony, and close kinship ties, with filial piety being deeply ingrained and profoundly shaping parent–child interaction patterns (Wang et al., 2014). Families in this context are characterized by a high degree of interdependence, and individuals tend to exhibit stronger emotional dependence on their families of origin (Chung and Gale, 2009; Wu, 2013). A survey targeting Chinese college students revealed that 25.8% of individuals with low levels of differentiation of self-exhibited pronounced emotional fusion features, scoring significantly higher on the “fusion with others” subdimension (Wu, 2010; Li et al., 2023). Even when college students in early adulthood become physically separated from their families of origin, the long-term impact of family functioning continues to persist. The family interaction patterns internalized during individual developmental stages consistently shape their self-perception, emotional regulation strategies, and approaches to environmental adaptation (Wang et al., 2014).

### 4.3 The independent mediating effects of loneliness and psychological security

Loneliness Serves as a Mediating Role Between Family Functioning and Sense of Security. Large-sample surveys have shown that the detection rate of loneliness among young adults can reach 58% (Flesaker et al., 2025), indicating that loneliness is a prevalent negative emotional experience among college students. As an unpleasant experience, loneliness arises when individuals perceive that their social relationships do not meet expectations and when there is a lack of interpersonal contact. Loneliness is significantly positively correlated with individuals' mental health (Tian et al., 2014; Jiang et al., 2017). Individuals with high levels of loneliness are more likely to adopt social withdrawal strategies, such as reducing interpersonal interactions, which hinders the development of self-differentiation. The diathesis-stress model suggests that highly vulnerable individuals in low-family-functioning environments are prone to developing maladaptive patterns (Zhou et al., 2016). When maintaining greater distance from others, individuals experience intense loneliness and are more likely to employ psychological defense mechanisms such as rejection, denial, and intellectualization to reduce communication with others. When individuals remain in this state for an extended period, it becomes difficult for them to develop the capacity for self-differentiation. Conversely, individuals with low levels of self-differentiation tend to rely on others in interpersonal interactions, and their thoughts and emotions are easily influenced by others.

Sense of security as a key psychological mechanism mediating the relationship between family functioning and differentiation of self. When college students perceive their environment as predictable and believe they possess the ability to cope with interpersonal challenges and academic stress, they develop a stable sense of psychological security (Yang et al., 2025). According to Self-Determination Theory, individuals with a stronger sense of security are more likely to make behavioral choices based on intrinsic psychological needs (e.g., autonomy, competence, and relatedness) rather than external pressures, thereby demonstrating higher psychological adaptability and proactivity (An and Chen, 2015). In conjunction with Belsky's differential-susceptibility model, highly sensitive individuals exhibit bidirectional responses to family environment influences: in negative environments, low family functioning leads to a deficit in sense of security and a reduction in Differentiation of Self levels; in positive environments, highly sensitive individuals can significantly enhance their differentiation capacity through the mediating role of sense of security when receiving moderate levels of support (Zhang et al., 2015). High-functioning families (high emotional support, low conflict) promote family members' experiences of security by providing a “safe base”; low-functioning families (e.g., high control, low responsiveness), in contrast, undermine sense of security through the transmission of inter-generational anxiety (Zwane, 2012). It is worth noting that one indirect pathway (Family Functioning → Security → Differentiation of Self), though statistically significant, yielded a negligible standardized effect estimate of −0.023. This finding warrants cautious interpretation, as statistical significance does not necessarily imply practical meaningfulness, particularly in the context of a large-sample design where even trivial effects may reach conventional levels of significance.

### 4.4 Serial mediating effects of loneliness and psychological security and gender differences

The chain mediation model of loneliness and sense of security was validated (Hypothesis 3 supported).The χ^2^ test is highly sensitive to large sample sizes and should not be interpreted in isolation. Conventional cut-off values were adopted: CFI and TLI > 0.90 indicate acceptable fit; RMSEA below 0.08 and SRMR below 0.08 represent adequate model fit. Loneliness undermines individuals' cognitive appraisal of environmental controllability, leading to a decrease in sense of security (An and Chen, 2015; Wen et al., 2021). When college students receive stable emotional support and their needs for social connectedness during the environmental adaptation period, loneliness levels significantly decrease (Wang et al., 2014), and social avoidance behaviors are reduced by 37% (Wang et al., 2014). College students with adaptive advantages exhibit stronger prefrontal regulation over the amygdala and lower stress responses (e.g., cortisol levels) (Wang et al., 2021). Upon entering college and detaching from original family attachment relationships, college students experience a temporary gap in social connectedness resources, accompanied by a significant increase in loneliness (Jiang et al., 2017). According to the diathesis-stress model, family functioning deficits serve as stressors that trigger emotional experiences of loneliness and reduce individuals' sense of security regarding both internal and external environments (An and Chen, 2015). Prolonged high levels of loneliness persistently undermine individuals' perceived controllability over the external environment, making it difficult for college students to trust others and establish stable interpersonal relationships, thereby impairing their psychological security.

Loneliness as an emotional response to low family functioning exhibits cross-gender consistency, yet the subsequent psychological and behavioral divergences are gender-specific: when loneliness emerges, females are more likely to alter their self-stance due to interpersonal factors, whereas males are more prone to manifest emotional disconnection in relationships. At the level of adaptive behaviors, when individuals experience loneliness, they tend to adopt negative coping strategies such as social withdrawal and emotional disconnection to reduce persistent interpersonal frustration (Mason, 2024), further constricting their interpersonal interaction space and leading to a continued decline in sense of security, thereby forming a negative cycle of “low family functioning → elevated loneliness → insufficient sense of security.” According to Bowen's family systems theory, college students with low differentiation of self, due to their deficient emotion regulation capacity, are susceptible to amplified loneliness when families fail to provide adequate emotional support, which in turn disrupts interpersonal boundaries. Loneliness may be an emotional byproduct derived from dysfunction in the family-of-origin.

Differentiation of Self is a lifelong developmental task for individuals, with the college years constituting a critical formative period for its growth. Notably, the prevalent phenomenon of “post-Gaokao divorce” in Chinese families reflects latent risks associated with inter-generational emotional transmission (Liu et al., 2015). Parents who sustain superficial family harmony while concealing internal conflicts expose their children to a long-term environment of emotional inauthenticity and illusory stability, hindering the development of children's healthy emotional recognition abilities. College students raised in such family contexts tend to exhibit polarized patterns in intimate relationships, manifested as either excessive emotional fusion (e.g., people-pleasing tendencies) or defensive emotional cutoff (Deng et al., 2015). Universities should recognize that campus social interventions may inadvertently foster pseudo-differentiation among college students. Specifically, after entering university, students may rely on peer relationships to compensate for inadequate emotional support from their families. This state creates a superficial sense of independence and clear interpersonal boundaries, yet it fails to facilitate genuine cognitive and emotional separation at the internal level. To mitigate college students' loneliness, it is essential to optimize family functioning, facilitate positive intra-family emotional communication, enhance students' psychological security, and ultimately promote the healthy development of Differentiation of Self.

## 5 Conclusions

Family functioning significantly predicts loneliness and psychological security, which in turn exert cascading effects on college students' differentiation of self. As young adults, college students possess well-developed learning and logical reasoning capacities, allowing them to engage in self-regulation through heightened self-awareness and self-understanding. Low family functioning positively predicts loneliness while negatively predicting differentiation of self. In contrast, favorable family functioning positively predicts both psychological security and differentiation of self. These findings underscore the need for mental health education to prioritize the alleviation of loneliness, the enhancement of psychological security, and the promotion of differentiation of self among college students.

## 6 Limitations and future prospects

This study examined the influence of family functioning on college students' differentiation of self. Although the research was grounded in both theoretical deduction and empirical evidence, utilized well-established scales with sound psychometric properties, and employed appropriate statistical analyses, the cross-sectional design imposes inherent limitations. Accordingly, future research would benefit from adopting longitudinal designs and more rigorous methodologies to enable deeper inquiry. Guided by Bowen's Family Systems Theory, the present study explored how loneliness and psychological security mediate the relationship between family functioning and differentiation of self during the transition to university. Subsequent investigations might also consider testing the reverse pathway—specifically, whether differentiation of self can enhance psychological security and alleviate loneliness—as well as its broader implications for positive emotional experiences throughout the college years. Moreover, all participants were recruited exclusively within China, and the observed associations are embedded in the Chinese cultural context. Given that cultural values shape family interaction norms, self-construal, and the developmental features of differentiation of self, future work could conduct cross-cultural and comparative studies to determine whether the serial mediation model identified here possesses universal applicability or exhibits culturally specific variations.

## Data Availability

The original contributions presented in the study are included in the article/supplementary material, further inquiries can be directed to the corresponding author.
